# Evaluation of immunomodulatory and antioxidants properties of *Kwath,* conventional extracts in plants *Cocculus hirsutus* and *Cuscuta reflexa* – *in vitro* & *ex vivo* studies

**DOI:** 10.1016/j.jaim.2021.100537

**Published:** 2022-01-10

**Authors:** Anita Singh, Vandana Singh, R. Ananthan, B. Dinesh Kumar

**Affiliations:** a Food and Drug Toxicology Research Centre, National Institute of Nutrition (ICMR), Jamai Osmania, Hyderabad, 500 007, India; b Food Chemistry Division, National Institute of Nutrition (ICMR), Jamai Osmania, Hyderabad, 500 007, India

**Keywords:** Immunomodulation, Ethnic food, Unexplored medicinal plants, Th1/Th2 cytokines, Nutraceuticals, UHPLC, Ultra High Performance Liquid Chromatography, DPPH, 2,2-diphenyl-1-picrylhydrazyl, ABTS, 2,2-azino-bis-3-ethylbenzthiazoline-6-sulphonic acid, LPS, Lipopolysaccharide, ROS, Reactive oxygen species, Con A, Concanavalin A, K, *Kwath*, Eth, Ethanolic, HE, Hydroethanolic

## Abstract

**Introduction:**

The consumption of ‘*Patalagarudi*’ (*Cocculus hirsutus* ‘CHP’) and ‘*Amarbel*’ (*Cuscuta reflexa* ‘CRA’) as ethnic plants for health promotions rarely validated. The limited literature reported these plants as antioxidant and immunomodulators.

**Objective:**

To evaluate the biodynamic properties of CHP and CRA extracts.

**Methodology:**

The traditional formulation, ‘*Kwath*’ (K) and conventional extracts were prepared with CRA and CHP. The total phenolic content (TPC) was estimated. Various polyphenol compounds in the extracts were eluted on UHPLC. The biodynamic activities; i. Free radical scavenging (FRS-DPPH and ABTS), ii. Intracellular ROS scavenging activity in RAW 264.7 cell line iii. Spleenocytes proliferation assay for Th1/Th2 Immunomodulatory potential by flow-cytometer were assessed.

**Results:**

The TPC in CRA (105–159 μg GAE/mg) and CHP (35–48 μg GAE/mg) recorded. The chromatographic peaks confirmed the presence of polyphenols in CRA and CHP extracts. UV spectra of the extracts to the extent possible have been correlated with certain polyphenols. The FRS (IC50) was significantly low in CRA-*K* (DPPH = 22.7; ABTS = 12.0 μg/ml) than CHP-*K* (DPPH = 70.4; ABTS = 50.2 μg/ml). Similarly, intracellular ROS scavenging activity with CRA-*K* (84%) showed the highest inhibitory potential compared to CHP-*K* (50%) and LPS control. The immunomodulatory activity of CRA-*K* significantly upregulated TH1 cytokines (TNFα and IFN-γ). The downregulation of Th2 cytokines (IL-4 and IL-10) was in all CRA and CHP extracts as compared to Con A.

**Conclusion:**

The current study confirms the immunomodulatory and antioxidant properties of CRA and CHP along with the presence of polyphenols.

## 1 Introduction

The traditional knowledge of food, medicinal plants for improving health is gaining much significance. For several decades, the documentation of indigenous knowledge into health benefit practices is being translated to make the value addition of the products [1,2]. The *Ayurveda* Materia Medica has documented *Rasayana* (antioxidant), *Balya* (immunomodulatory), *Sheet Veerya* (anti-inflammatory), *Yakridvikarhrit* (liver protective) potential activity of *Asparagus racemosus, Tinospora cordifolia, Andrographis paniculate, Murraya koenigii, Ocimum sanctum, Terminalia arjuna, Piper longum, Moringa oleifera* in clinical practice [3].

The ethnic population consumes over 85% of lesser-known plants based on knowledge/experience of previous generations. *Cocculus hirsutus, Cuscuta reflexa* are lesser-known/neglected plants of some ethnic communities reported to have food/medicinal properties [[4], [5], [6], [7]]. However, some ethnic plants, which have confined uses, are rarely analyzed and correlated to health benefits [[8], [9]]. The validations of such knowledge contribute to value addition as well as give a scope to list them as certified medicinal plants.

The “*Patalagarudi*” (botanical name *C. hirsutus* (CHP)) is a green leafy vegetable and is consumed in the southern Indian ethnic group [6,7]. The available information suggests its role in wound healing, tuberculosis, skin diseases and as a cooling agent [10,11]. The traditional text described their role as *Vishdodha* *Vinashini* (anti-toxin and help to maintain homeostasis in the body) and *Vrisya* (good aphrodisiac) [3,12]. Some of the pharmacological activities such as anti-inflammatory, anti-diabetic, anti-microbial, anti-cancer, hepatoprotective, immunomodulatory were also validated in the experimental models [10,11,13].

The ‘*Amarbel*’ is popular in local folk of the hilly area of northern India for use against snakebite, wound healing. Its botanical name is *C. reflexa* (CRA) which is traditionally used to treat conjunctivitis, respiratory disorders, piles, ulcers, and stomach problems [14]. *Cuscuta* species are commonly used as herbal constituents in functional foods and medicinal tonics to nourish the different body parts [5]. The traditional text also describes the therapeutic role of CRA as *Aamnashini* (elimination of oxidative stress), *Agnikari* (ameliorating metabolic activity), *Hrdya* (cardio protective) and *Yakshmapaha* (anti-tuberculosis) [12]. The pharmacological role of CRA has been identified as an anti-inflammatory, anti- HIV, anti-osteoporotic, anti-tumor, antioxidant, anti-osteoporotic [4,[13], [14], [15], [16]]. Many researchers have shown that plants contain appreciable quality and quantity of alkaloids, polysaccharides, steroids, terpenoids, flavonoids, amino acids, fatty acids, and antibiotics, which are responsible for their pharmacological role [[17], [18], [19], [20], [21]]. Since few decades our institute is validating ethnic food/medicinal plants for their immunomodulatory property, antioxidant, anti-inflammatory and other pharmacology activity [[22], [23], [24], [25], [26], [27]]. Among several studies, inflammation-induced oxidative stress and immunomodulatory activity of the plants through Th1 (TNF-α and IFN- γ)/Th2 (IL-4 and IL-10) cytokines have been undertaken [[22], [23], [24]]. Various studies have investigated the immunomodulatory activity of the plants using Th1 and Th2 cytokines as bio-makers in *ex vivo* and *in vivo* models [[22], [23], [24], [25],[28], [29], [30]]. Chendouh et al., 2019 have reported *Inula viscosa* (unexplored plant) can be a potential nutraceutical due to the presence of forty-three secondary metabolites with remarkable antiradical and cytotoxic activity [31]. In the present study, the consumption pattern of CHP and CRA in different forms along with support of traditional knowledge have been identified to evaluate various biological activities. Therefore, the objective of the study was to assess antioxidant and immunomodulatory potential of CHP and CRA using *in vitro* and *ex vivo* model respectively. In addition, the composition analysis, total phenolic content (TPC), and determining the presence of polyphenols in various extracts of CHP and CRA were performed.

## 2 Materials and methods

### 2.1 Reagents

ABTS (2,2-azino-bis-3-ethylbenzthiazoline-6-sulphonic acid), DPPH (2,2-diphenyl-1-picrylhydrazyl), Ascorbic acid, DCFDA (2,7-Dichlorofluorescein diacetate), LPS (Lipopolysaccharide) isolated from *Escherichia coli* 055:B5, Gallic acid and RPMI medium were purchased from Sigma Chemical Co. (St. Louis, MO, USA). Folin–Ciocalteu's phenol reagent, DMEM (Dulbecco's Modified Eagle Medium), FBS (fetal bovine serum), penicillin-streptomycin, Trypsin, MTT (3-(4,5-dimethylthiazol-2-yl)-2,5-diphenyl tetrazolium bromide), Con -A (concanavalin A) were purchased from HI media. Mouse Cytometric bead array (CBA) immunoassay kit (Catalogue no. 558266) containing Th1/Th2 (TNFα, IFN-γ, IL-4, and IL-10) cytokines were procured from BD (Becton, Dickinson and Company, USA). RAW 264.7 macrophages cell line was purchased from the American Type Culture Collection (Rockville, MD). All chemicals used were of analytical grade.

### 2.2 Plant selection

The medicinal plant (CRA) and ethnically consumed food (CHP) were collected from a local farm breeder. These plants were identified by a taxonomist, herbariums were prepared and deposited at local Botanical Survey of India centers.

#### 2.2.1 Parts of the plant

The aerial part of CRA was collected by a local breeder, dried in sunlight for 8–10 days. The leaves of CHP were collected by the investigators and dried in sunlight for 4–5 days. The dried plants materials were powdered using a cutting mill (Retsch SM 100, Germany) and sieved using sieve of aperture size 0.5 mm. The resulting powders were used for the following analysis.

#### 2.2.2 Compositional analysis

The compositional analysis includes macronutrients content (carbohydrates, proteins and fats) and micronutrient content (vitamin B2, B3, B5, B6 and C), minerals (Phosphorus, Magnesium, Potassium, Iron, Zinc, Copper, Manganese, Selenium, Molybdenum, and Chromium) and plant sterols (β-sitosterol, stigmasterol, Campesterol) were analyzed by standard AOAC (Association of Official Agricultural Chemists) methods at Food chemistry department, ICMR-NIN [32].

### 2.3 Extracts preparation

#### 2.3.1 Traditional *Kwath* extract preparation

Powdered plant material (5 g) was soaked in 100 ml of MilliQ water for 60 min and then heated at 50 °C to reduce the volume to 25 ml. This extract was lyophilized (Scanvac cool safe 110-4, Denmark) and used in subsequent experiments.

#### 2.3.2 Conventional extracts

These were prepared in ethanolic and hydroethanolic solvents.

##### 2.3.2.1 Ethanolic extract

This was prepared by soaking the powdered materials (5 g) in 100 ml of absolute ethanol for 48 h with continuas shaking. The extract obtained was evaporated using rotavapor (BuchiR-205 Rotavapor System, Switzerland). The percent yield of the extracts was calculated. The lyophilized extract was stored at −20 °C.

##### 2.3.2.2 Hydroethanolic

This was prepared by soaking the powdered materials (5 g) in 100 ml of hydroethanolic solution (ethanol: water = 1:1) for 48 h on a shaker. It was followed by evaporation using rotavapor (Buchi R-205 Rotavapor System, Switzerland) and subsequent extract was lyophilized. The percent yield of the extracts was calculated. The lyophilized extract was stored at −20 °C.

### 2.4 Determination of polyphenol

#### 2.4.1 Total phenolic content (TPC)

The phenolic content was determined by Folin–Ciocalteau reagent using Gallic acid as a standard [33]. The total phenolic content of the extract was determined with a standard curve method using gallic acid in different concentrations (20–100 μg/μl). The absorbance was measured at 725 nm by a spectrophotometer. The final concentration of the total phenolic content present in the extracts was expressed as μg of Gallic Acid Equivalents (GAEs).

#### 2.4.2 Monitoring polyphenols compounds

The extracted samples of CRA and CHP plants were subjected to analysis for polyphenols on Ultra High performance liquid chromatography coupled with UV/Visible, fluorescent Spectrophotometry (DIONEX; UHPLC Ultimate 3000 Thermo Scientific) following standard methodology [32,34].

All lyophilized samples were dissolved in DMSO at 1 mg/ml concentration. These were filtered through a 0.22 μm filter, before injecting (5 μL) into UHPLC system. The C18 100mmX2.1 mm, .21 μm, 120Ao, Acclaim, RSLC PA2 column maintained at 35 °C was used. The solvent system included 90% Phosphate buffer (50 mM pH 3.3), 10% methanol as eluent A, 30% Phosphate buffer (50 mM pH 3.3) and 70% methanol as eluent B, delivered at a flow rate of 0.47 ml/min as follows: initially 100% of solution A; for next 2.640 min, 70% A; for another 5.28 min, 65% A; for another 3.52 min, 60% A; for another 0.88 min, 50% A; for another 5.08 min, 0% A and finally 100% A for 4.6 min. The online chromatogram was monitored at 250, 280,320 and 370 nm. The area of auto-integrated chromatography peaks of different polyphenols for reference compounds as well as samples were documented.

A mixture of standard polyphenols (Gallic acid, Protocatechuic acid, Chlorogenic acid, Vanillic acid, p-hydroxy benzoic acid, Caffeic acid, Sinapic acid, Ferulic acid, 4-Coumaric acid, 2-Coumaric acid, Daidzein, Flavones &Apigenin, Catechin, Luteolin7OGlucoside, Ellagic acid, Myricetin, Hesperetin, Quercetin, Luteolin, Kaempferol, Bergenin) was used as reference. The above stock solution was freshly prepared in Dimethyl sulfoxide (DMSO) at a concentration of 25 μg/ml. All the extracts of CRA and CHP viz., *Kwath* (K), Ethanolic (Eth) and Hydroethanolic (HE) were subjected to UHPLC analysis. We have abbreviated the presence of Polyphenols as “P” (present) followed by eluted peaks as “S” (strong), “M” (Medium), “L” (Low), and “T” (Trace). This HPLC data were correlated with the UV absorption data.

### 2.5 Free radical scavenging (FRS) activity

The free radical scavenging activity was determined by the following:

#### 2.5.1 DPPH (2,2-diphenyl-1-picrylhydrazyl) assay

The percentage DPPH radical scavenging activity was determined by Blois method (1958) [35]. Briefly, a stock solution (1 mg/ml) was prepared in Milli-Q water with each plant extracts (*Kwath*, Eth and HE). In 96 well plate, 150 μL of plant extracts (at different concentrations) was added along with 50 μL of DPPH solution (0.2 mM in methanol). The control was DPPH solution with methanol. Ascorbic acid was used as standard. The plate was allowed to rest in dark at room temperature for 30 min. The absorbance was recorded at 540 nm using a multimode reader (Synergy H1 hybrid reader; Biotek, Canada). The percentage free radical scavenging activity was calculated using the following formula;

$$\text{Percentage}\;\text{DPPH}\;\text{radical}\;\text{scavenging}\;\text{activity}=\frac{[\left(\text{Absorbance}\;\text{of}\;\text{control}-\text{Absorbance}\;\text{of}\;\text{test}\;\text{sample}\right)\times 100}{\left(\text{Absorbance}\;\text{of }\;\text{control}\right)}$$

The IC50 values were calculated based on the line graph representing the percentage of DPPH radical scavenging activity versus concentrations of the respective plant extracts/standard.

#### 2.5.2 ABTS (2,2-azino-bis-3-ethylbenzthiazoline-6-sulphonic acid) assay

The ABTS free radical scavenging activity of different plants extracts was determined by following Mir et al., 2018 method [36]. Briefly, to generate ABTS+, ABTS (7 mM) and potassium persulfate (2.45 mM) were incubated in the dark at room temperature for 16 h. The freshly prepared ABTS + solution was diluted with ethanol to obtain an absorbance at 734 nm of 0.70 ± 0.02. Ascorbic acid was used as standard. The reactive mixture along with samples was placed in the dark at room temperature for 15 min. The absorbance was recorded at 734 nm using a multimode reader (Synergy H1 hybrid reader; Biotek, Canada). The percentage ABTS radical scavenging activity was calculated using the following equation:

$$\text{Percentage}\;\text{ABTS}\;\text{radical}\;\text{scavenging}\;\text{activity}=\frac{[\left(\text{Absorbance}\;\text{of}\;\text{control}-\text{Absorbance}\;\text{of}\;\text{test}\;\text{sample}\right)\times 100}{\left(\text{Absorbance}\;\text{of}\;\text{control}\right)}$$

The IC50 was calculated from the ABTS scavenging activity (%) versus concentrations of the respective plants extracts/standard.

#### 2.5.3 Intracellular ROS scavenging activity in RAW 264.7 macrophage cell line

**Fig. 2:**
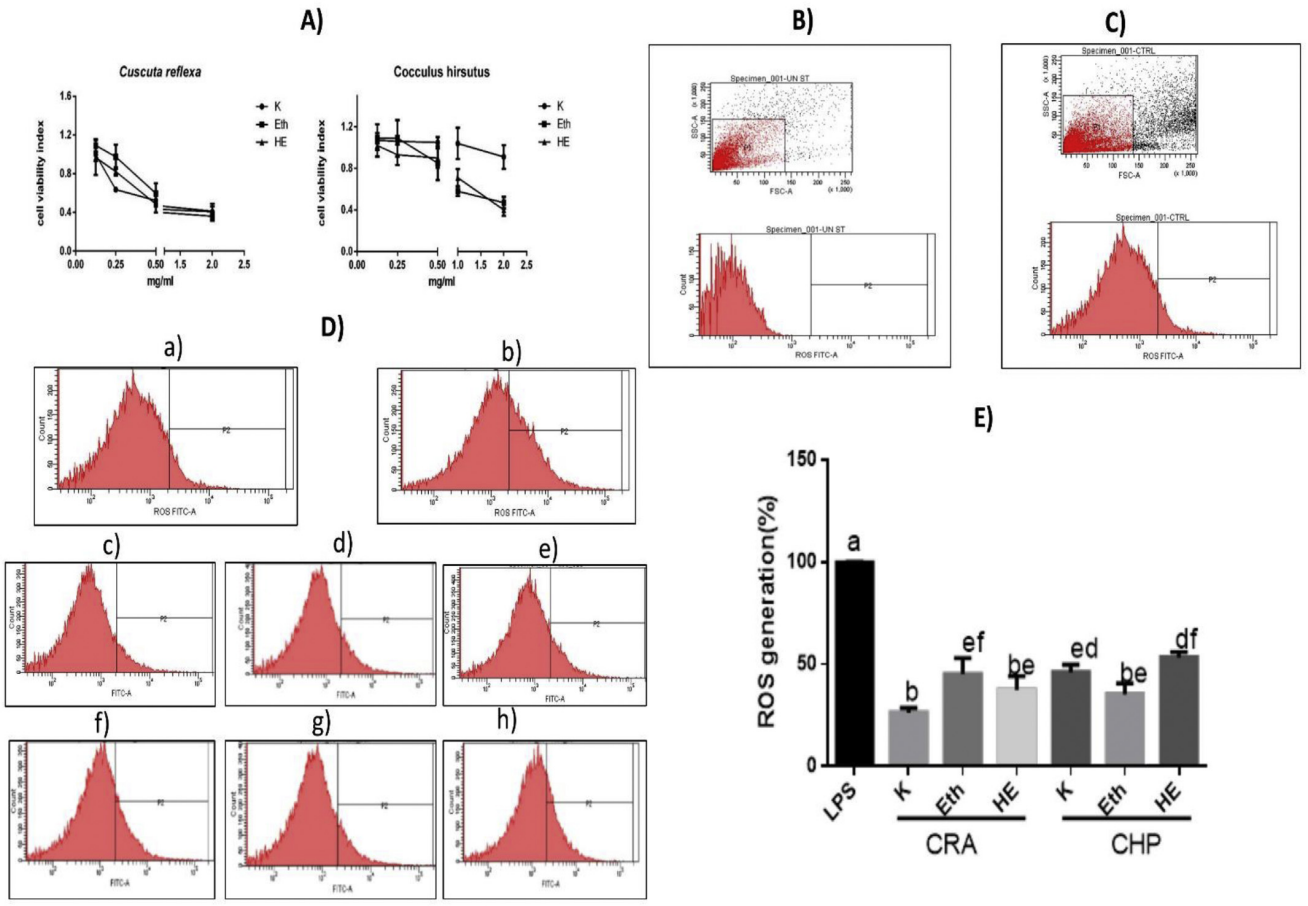
Effect of different plants extracts on the cell viability and intracellular ROS scavenging activity in RAW 264.7 macrophages. A) The cell viability was assessed using an MTT reduction assay and the results are expressed as the cell viability index compared with the control group (no extract and LPS treatment). Values are expressed as the mean ± SD of the three independent experiments. LPS-induced ROS generation in RAW 264.7 macrophages was measured using flowcytometry using excitation and emission wavelengths of 485 and 535 nm, respectively. B) unstained control (without DCFDA) C) negative control; stained (with DCFDA) (D) The representative graphs of ROS generation; a) negative control (no LPS treatment) b) LPS (500ng/mL); positive control (c) CRA(*Kwath*) d) CRA(Eth) e) CRA(HE) f) CHP(*Kwath*) g) CHP(Eth) h) CHP(HE). (E) Quantified levels of ROS. Values are expressed as the mean ± standard deviation. Different superscripts imply statistical significance P < 0.05. ROS, reactive oxygen species, LPS, lipopolysaccharide.

The inhibition of intracellular reactive oxygen species (ROS) was determined using a previously published method [37] with slight modification. It includes standardization of viability of cells in the presence of different concentrations of extracts (0.125–2 mg/ml) and LPS (500 ng/ml) using MTT (3-(4,5-dimethylthiazol-2-yl)-2,5-diphenyl tetrazolium bromide) assay. For the oxidative stress assay, the RAW 264.7 macrophages (2 × 10^6^ cells/ml) were allowed to grow in DMEM medium along with extracts at the effective concentration (0.125–2 mg/ml) in 24 well cell culture plate. Subsequently, LPS was added to each well except negative control to induce ROS formation. After 24 h of incubation, cells were washed with sterile PBS, trypsinized and collected in dark tubes. The residue trypsin was removed by washing the cells again with sterile PBS. The intracellular ROS formation was estimated using ROS-sensitive fluorescent dye, DCFDA (2,7-Dichlorofluorescein diacetate) in macrophages. The DCFDA (final concentration 10 μl of 10 μM) was added to each tube and incubated for 30 min at 37 °C and the fluorescence intensity of the stained cells was determined using a flow cytometer instrument (BD, FACSAria-II Cell Sorter, USA) utilizing excitation and emission wavelengths of 485 and 535 nm, respectively. Briefly, forward vs. side scatter gating was employed to exclude any sample particles other than the main cell population. The gating on the cells of interest opens a histogram plot with FL1 (fluorescent dye, CM-H2DCFDA) on the x-axis. The marker gate was drawn to distinguish between unstained cells which appear on the left of the histogram and ROS (oxidative stress) was detected as a shift of cells to the right (Fig. 2B and C). The data analysis was performed in flow cytometer software (FACSDiva Software) where at least 10,000 cellular events were counted on FL1 (fluorescent dye, CM-H2DCFDA) channels.

### 2.6 *Ex vivo* immunomodulatory activity

This experiment was standardized utilizing the spleens from 10 mice (naïve healthy swiss albino female mice, 8- to 10-week-old, weighing 18–22 g). This was followed by optimization of procedures to evaluate individual plant effects by measuring spleenocyte proliferation and cytokine estimations. At the experimental phase, 3 spleens were harvested in RPMI 1640 medium and one of them has been teased to perform spleenocyte proliferation assay in triplicate. The institutional animal ethical committee approval was sought (P28F/III-IAEC/NIN/12/Swiss).

#### 2.6.1 Spleenocyte proliferation activity

Under the aseptic conditions, the spleen samples were teased using fine steel mesh to prepare homogenous single-cell suspension in RPMI 1640 medium. It was followed by three times washing of the spleen solution to obtain healthy cells pellet to ensure uniform distribution of cells in the suspension medium. Briefly, 2 × 10^6^ cells/ml were plated in each well, along with various plant extracts in concentrations ranging from 62.5 to 500 μg/ml in RPMI-1640 medium containing 10% FBS. The plates were incubated for 48 h at 37 °C which was followed by the addition of MTT (5 mg/ml). To test the stimulatory potential of extracts in the presence of T cell mitogen, Concanavalin A (5 μg/ml) was used. The various concentrations of plant extracts (62.5–500 μg/ml) in RPMI-1640 medium containing 10% FBS were incubated along with Con A for 48 h at 37 °C. Con A was used as a positive control. The proliferation capacity of the test compounds was determined by MTT method. The plates were read under a multimode spectrometer (Synergy H1 hybrid reader; Biotech, Canada) at 570 nm. The stimulative index (OD of treatment/OD of control) was calculated. Based on SI, the best immunostimulatory concentration of each plant extract was selected for further cytokines estimation.

#### 2.6.2 Cytokines estimation

Spleenocytes (approximately 2 × 10^6^ cells/ml) were treated with the best immunostimulatory dose of each plant extract (K, Eth and HE) with the concanavalin A (5 μg/ml) in 24 well plates. It was followed by incubation for 72 h at 37 °C. Next, cells were centrifuged for 5 min at 1500 rpm and the supernatant was collected for cytokines analysis using a flow cytometer. The Th1/Th2 (TNFα, IFN-γ, IL-4, IL-10) cytokines were determined in the supernatant using mouse Cytometric bead array (CBA) immunoassay kit (BD Bioscience, USA) according to the manufacturer's instructions. Briefly, each cytokine standard/supernatant was incubated with a mixture of capture antibodies-bead reagent and detector antibodies reagent which were conjugated to a reporter molecule (phycoerythrin; PE) to form sandwich complexes. The intensity of PE fluorescence of each sandwich complex reveals the concentration of that particular analyte. The data were acquired using BD FACSAria II. Forward vs. side scatter gating was employed to exclude any sample particles other than the cytokines beads. The different beads population were clustered using a two-color dot plot (APC-A vs APC-Cy7-A) across the x-axis and y-axis to measure the distinctive fluorescence characteristics of beads. The standard curves (0 pg/ml to 2500 pg/ml) were plotted for all four cytokines (concentration vs. mean fluorescence intensity; MFI) using a five-parameter logistic curve fitting model (Fig Supplementary(S) 1). The unknown concentration of cytokines in the cell culture supernatant was detected using these standard curves.

### 2.7 Statistical analysis

Results were presented as the mean of triplicate ± standard deviation (SD). Linear regression analysis was performed using GraphPad Prism 6.0 (GraphPad Software Inc., San Diego, CA) for antioxidant assays. One-Way analysis of variance was used to compare means, and the results were expressed as the mean ± SD. P-value <0.05 was considered statistically significant.

## 3 Results

### 3.1 Collection, identification, authentication and extractive yield

Both the plants (CRA and CHP) were identified by a taxonomist and collected from various regions based on their potential availability. The herbarium sheets were as prepared and bio-deposited with the Botanical Survey of India of Dehradun (CRA, ref no. 116103) and Hyderabad (CHP, ref no. 11522). The extractive yield in various solvents was constant for all individual plants. It was in the range of 10–33% and 9–33% for CRA and CHP respectively (Table 1).

**Table 1:** Extractive yield of CRA and CHP.

S.no	Test material	Extracts (g/100g)^a^		
		*Kwath*	Ethanolic	Hydroethanolic
1	*Cuscuta reflexa (CRA)*	33.33 ± 1.528	10.17 ± 1.258	27.50 ± 0.436
2	*Cocculus hirsutus (CHP)*	33.07 ± 0.306	9.00 ± 1.000	16.80 ± 1.709

a Data represents means ± SD of triplicates.

### 3.2 Compositional analysis

The compositional analysis of CRA and CHP demonstrated the presence of carbohydrates, proteins and fats, dietary fibers, Vitamins (B2, B3 and C) and minerals (Ca, K, P, Mg, Mn) (Table 2). CHP contains higher proportion of proteins (16.23%) and dietary fibers (48.59%) compared to CRA (9.4% and 32.8% resp.). The predominance of Riboflavin (2.83%), Niacin (4.84%) in CRA, and Vitamin C (26.26%) in CHP was recorded.

**Table 2:** Compositional analysis of CRA *and* CHP in 100 gm extract (equivalent to 500 gm of fresh samples).

Nutrients and minerals	*CRA*	*CHP*
Macronutrient (g)		
Carbohydrate	42.62	20.18
Protein	9.43	16.23
Fat	3.80	4.29
Dietary fiber(g)		
Insoluble dietary fiber	30.93	46.29
Soluble dietary fiber	1.85	2.3
Total dietary fiber	32.78	48.59
Water soluble vitamins (mg)		
B2	2.83	0.28
B3	4.84	2.81
B5	0.92	1.21
B6	0.32	0.29
Vitamin C	2.32	26.26
Minerals (mg)		
Phosphorus	407.22	341.77
Potassium	2313.03	1507.15
Magnesium	100.19	124.97
Iron	24.58	20.91
Zinc	1.39	1.62
Copper	0.79	1.33
Manganase	1.06	3.30
Minerals (μg)		
Selenium	3.16	10.94
Molybdenum	29.11	95.63
Chromium	96.97	79.07
Plants sterols (mg)		
B -sitosterol	47.77	42.84
stigmasterol	12.22	31.12
Campesterol	8.73	7.85

### 3.3 Determination of polyphenol

The TPC were in range of 105–159 μg GAE/mg in CRA extracts and 35–48 μg GAE/mg in CHP extracts (Table 3). The chromatograms and UV spectra for the above extracts along the reference phenolic compounds is given in (Fig Supplementary(S) 2 to 10).

**Table 3:** TPC of extracts of CRA and CHP using different solvents expressed in μg gallic acid equivalent/mg extracts.

Extract	CRA	CHP
*μg gallic acid equivalent/mg extracts*		
*Kwath*	158.54 ± 11.319^a^	47.63 ± 8.756^c^
Ethanolic	104.60 ± 5.068^b^	35.20 ± 6.013^d^
Hydroethanolic	156.80 ± 3.00^a^	36.57 ± 3.572^d^

Results are means ± SD of triplicate from 3 independent experiments. Values with different letters within the columns are significantly different (p < 0.05).

CRA extracts: The ellagic acid detected in UV spectra and eluted as a strong peak in UHPLC. The caffeic acid was detected in UV spectra of all extracts with presence of medium peak on UHPLC of Eth extract. Myceretin and Hespertin were also detected on UV spectra with low level peaks on UHPLC. The daidzein was observed as strong peaks in all extracts. The *Kwath* and HE extracts also showed the presence of bergenin as medium peak on UHPLC (Table 4, Fig S 4, 5, 6 & Fig S10 a, b, c).

**Table 4:** Polyphenols monitoring of CRA and CHP based on UHPLC retention time and UV spectra.

	Polyphenols	Reference compound		CRA						CHP					
				*Kwath*		Ethanolic		Hydro Ethanolic		*Kwath*		Ethanolic		Hydro Ethanolic	
		RT	UV							RT	UV	RT	UV	RT	UV
1	Caffeic acid	S	P		**P**	M	**P**		**P**	M		M	**P**	L	**P**
2	Sinapic acid	S	P	T		T		T	**P**	M		M		L	**P**
3	Ferulic acid	S	P	T	**P**					T				N	**P**
4	Myricetin	S	P					L	**P**	L		L			**P**
5	Quercetin	S	P							T	**P**	L		T	
6	Protocatechuic acid	S	P							S		S		S	
7	p-hydroxy benzoic acid	S	P							M	**P**	L	**P**	L	
8	Gallic acid	S	P							L		L		M	
9	Bergenin	S	P	M				M		L		L			
10	Chlorogenic acid	S	P			M				L		L	**P**		
11	4-Coumaric acid	S	P			M						T			
12	Ellagic acid	S	P	S	**P**	S	**P**	S	**P**						
13	Daidzein	S	P	S		S		S							
14	Hesperetin	S	P			L		L	**P**						

P = Present; S = Strong; M = Medium; L = Low; T = Trace; UV = Ultra violet-visible Absorption Spectra; RT = Retention Time.

**Note:** Compounds showing strong match for RT and UV spectra confirms the Presence **(P)** of phytomolecules. The rest of the phytomolecules were predicted to be present in CRA and CHP.

CHP extracts: The extracts had a strong peak corresponding to protocatechuic acid on UHPLC. Caffeic and sinapic acid were also found to be present on UHPLC. p-hydroxy benzoic acid was detected in the UV spectra in *Kwath* and Eth extracts and corresponding peaks were observed on UHPLC (M and L respectively) (Table 4; Fig S 7, 8, 9 & Fig S10 d, e, f).

### 3.4 Free radical scavenging (FRS) activity

#### 3.4.1 FRS- DPPH (2,2-diphenyl-1-picrylhydrazyl) assay

**Fig. 1:**
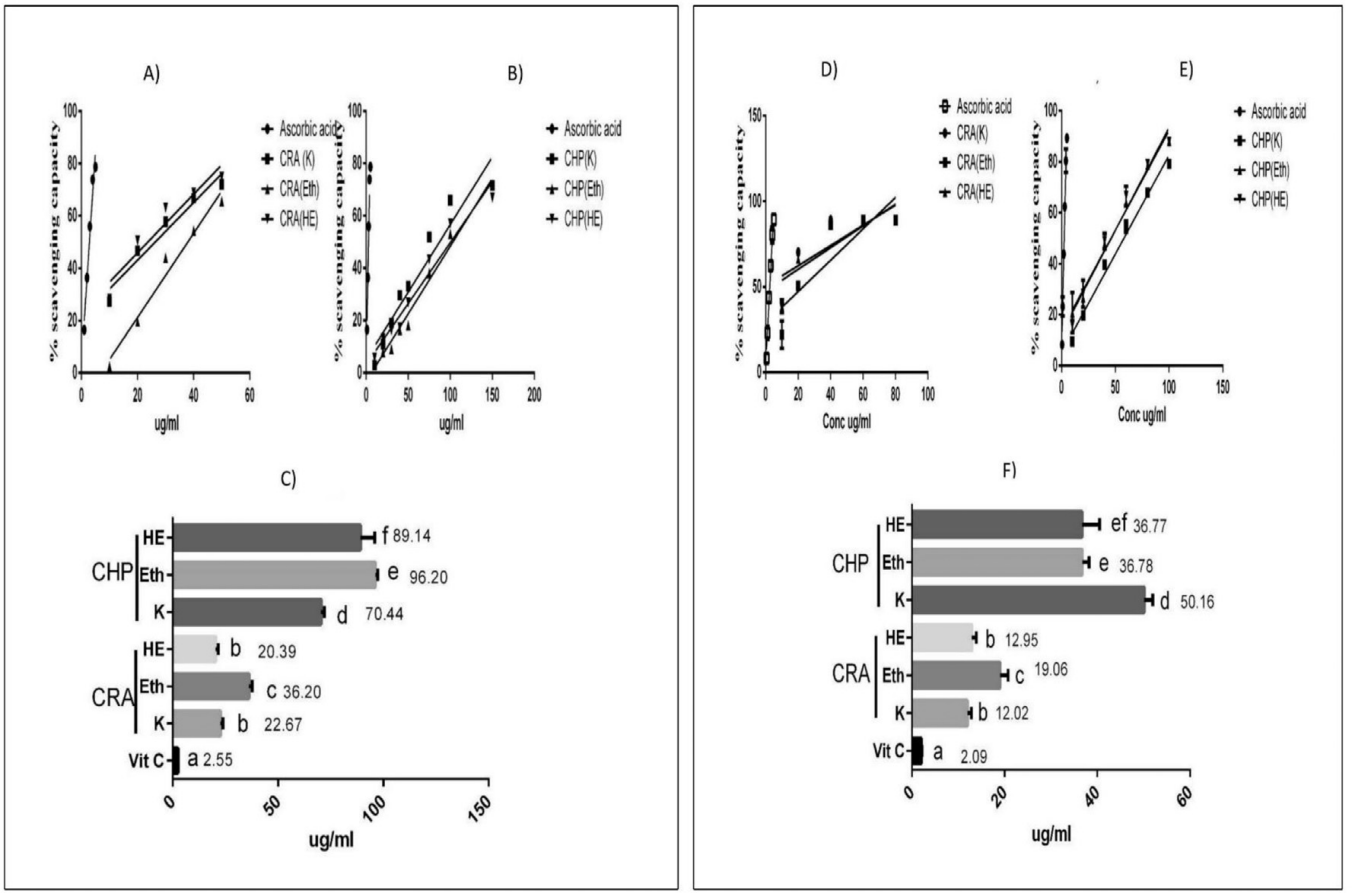
Effect of plant extracts on Free Radicals Scavenging (FRS) activity. DPPH scavenging capacities of A) CRA extracts and B) CHP extracts with Standard (ascorbic acid). ABTS scavenging capacities of D) *CRA* extracts and E) CHP extracts with Standard (ascorbic acid). X axis represents different concentration of plants in µg/ml; Y axis represents percentage scavenging activity. Results are means ± SD (n = 3). C and F represent IC50 values in the DPPH and ABTS assays respectively in *Kwath*, Eth and HE extracts of CRA and CHP. The IC50 values are expressed as mean±SD from three experiments (n = 3). Values with different letters within the same assay are significantly different (p< 0.05). DPPH = 2,2-diphenylpicrylhydrazyl radicals, ABTS = 2,2′-azino-bis diammonium salt radicals, the lower IC50 (μg/mL) the higher the antioxidant capacity. Bars having different letters are significantly different (*P* < 0.05).

The dose-dependent (10.0–1500 μg/ml) response between the increasing concentrations of individual extracts and % free radical scavenging activity of CRA and CHP was recorded. The IC50 of standard, Ascorbic acid was 2.5 ug/ml. The IC50 of CRA extracts was in the lower range (20–36 μg/ml) compared to CHP extracts (70–96 μg/ml). The concentration required by CRA extracts to scavenge the free radical (IC50) was found to be approximately 2–3 folds lesser than CHP, indicating CRA is more potent than CHP extracts (Fig. 1A–C).

#### 3.4.2 FRS-ABTS (2,2-azino-bis-3-ethylbenzthiazoline-6-sulphonic acid) assay

In the ABTS scavenging activities, the IC50 was found to be lower in CRA (12–19 μg/ml) compared to CHP (37–50 μg/ml) in all extracts. The IC50 of standard, Ascorbic acid was 2.09 μg/ml (Fig. 1D–F).

#### 3.4.3 Intracellular ROS scavenging activity in RAW 264.7 macrophage cell line

The cell viability (80%) with different extracts was standardized with CRA (0.125–0.25 mg/ml) and CHP (0.5–2 mg/ml) extracts (Fig. 2A). The inhibition of ROS generation was found to be ranging between approx. 20–85% with various extracts. The pretreatment with different extract *Kwath,* Eth, and HE extract of CRA and CHP significantly (*p* < 0.05) inhibited the Lipopolysaccharide (LPS)-induced ROS as compared to LPS control. The *Kwath* of CRA showed maximum ROS inhibition (84%) as compared to LPS control (*p* < 0.05) (Fig. 2).

### 3.5 Immune stimulation by CRA and CHP

**Fig. 3:**
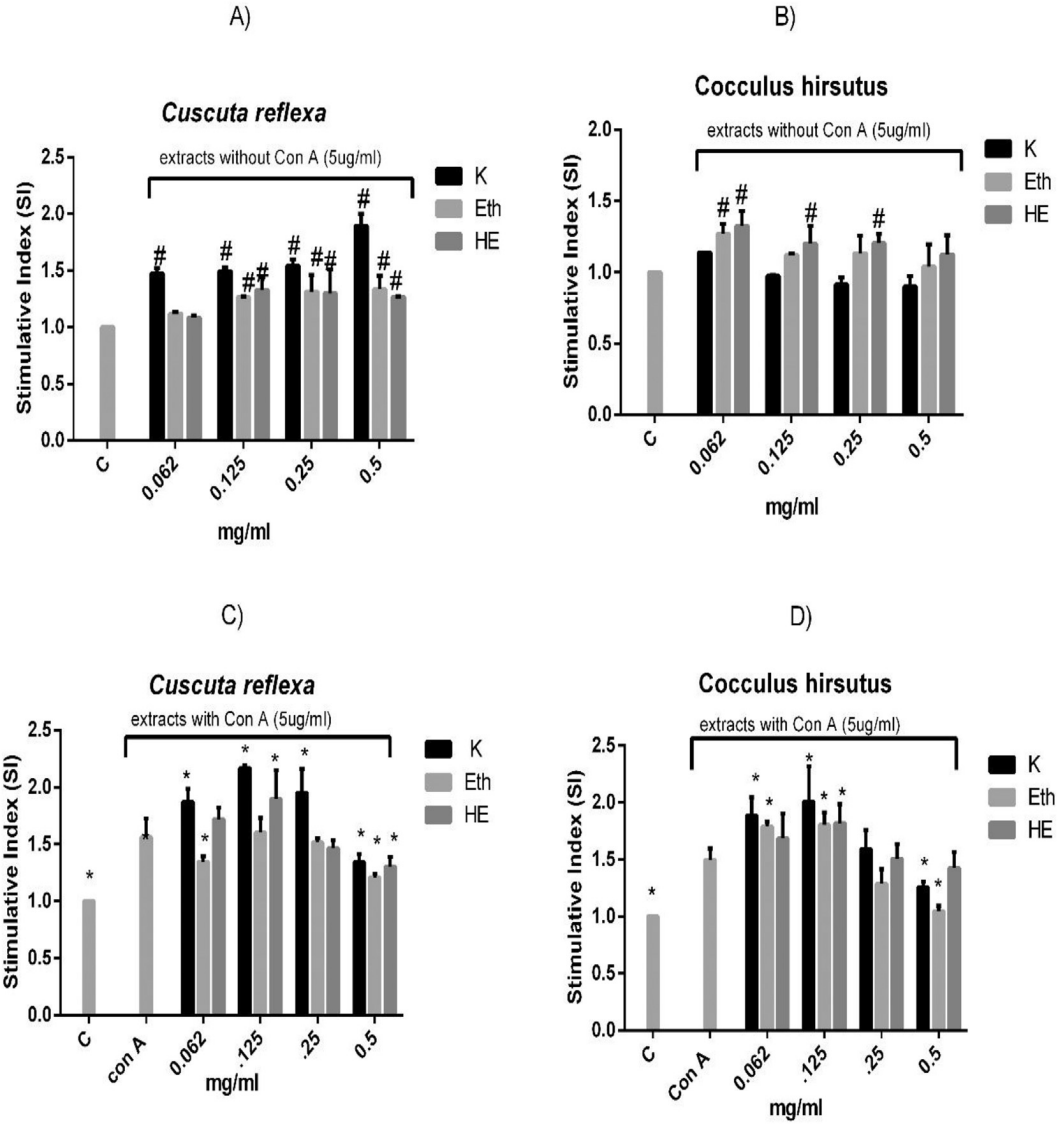
Effect of CRA and CHP extracts on *ex vivo* proliferation of spleenocytes. All data are presented as the mean ± SD. Data is expressed as Stimulative index (OD of treatment/OD of control). Hash mark (#) indicate significant difference (p ≤0.05) from Control (C). Asterisks (∗) indicate significant difference (p ≤0.05) from Con A.

The extracts of CRA and CHP demonstrated the ability to induce proliferation at various concentrations (0.0625–0.5 mg/ml) in unstimulated spleen cells. Furthermore, the plant extracts were co-stimulated with Con-A mitogen (T -cell lymphocyte proliferator) confirming the immunostimulatory effect of the extracts. Mitogen treatment resulted in a significant increase in cell proliferation (*p* < 0.05). The maximum mitogen (Con A) - induced spleenocyte proliferation was observed at 125 μg/ml in CRA *Kwath* and CRA HE extracts as compared to Con A (*p* < 0.05). The CRA Eth extract showed a narrow stimulatory index (SI) of 0.125 mg/ml although it was not significant. At the same concentration, CHP showed potential SI as compared to Con-A (*p* < 0.05) (Fig. 3).

**Fig. 4:**
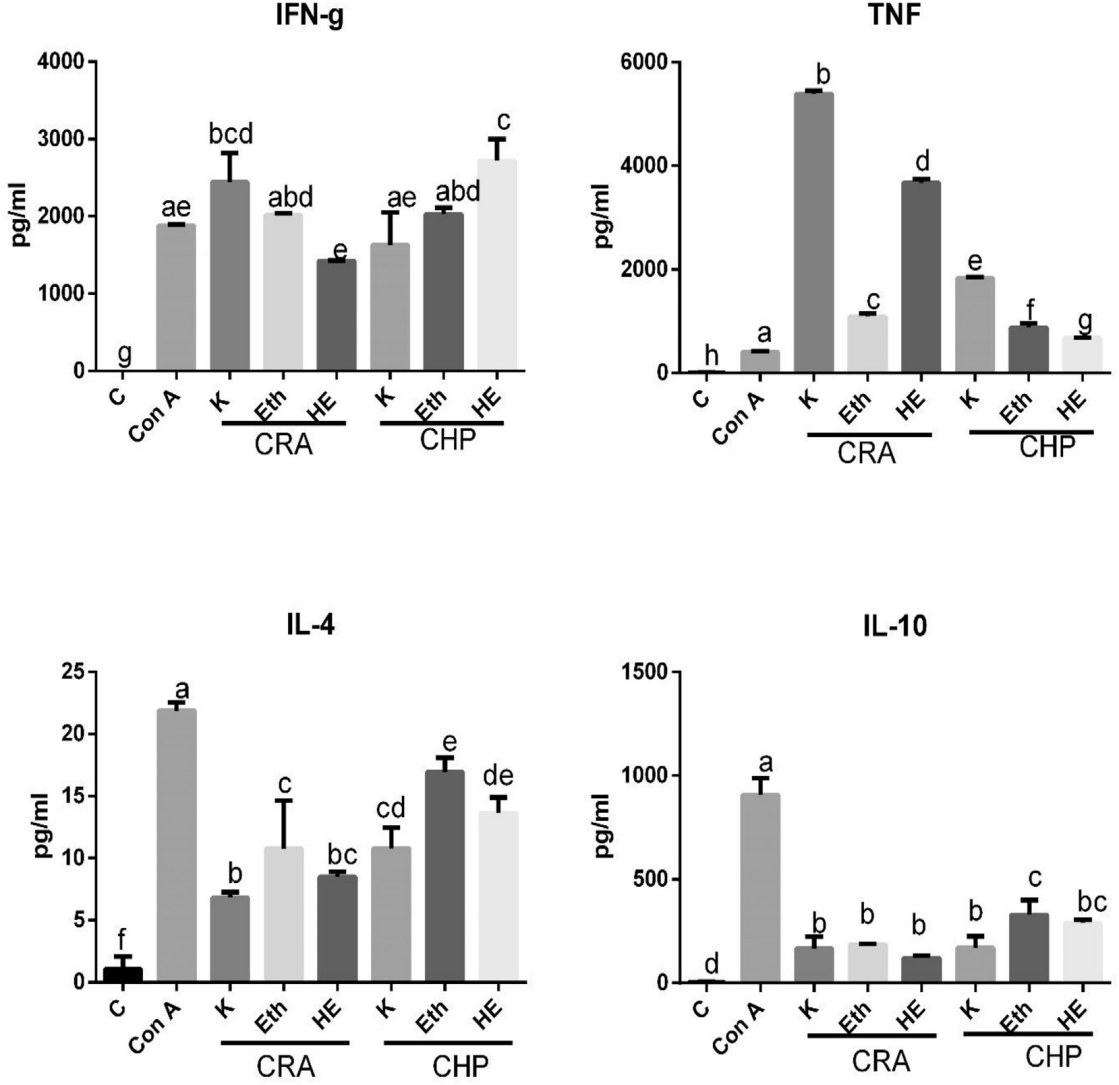
Effect of a) CRA extracts b) CHP extracts on splenic T lymphocytes cytokines. The levels of TH1/TH2 cytokines (in pg/ml; TNFα and IFN-γ / IL-4 and IL-10) were analyzed in supernatant of the spleenocytes culture in the presence of plants extracts and Con A. Cytokine levels were quantified by FACS. Experiments were performed in duplicate. Data shown are mean ± SD. Bars having different letters are significantly different (*P* < 0.05).

The above-mentioned concentrations were further selected to evaluate *in vitro* Th1/Th2 polarization (Fig. 4). The CRA *Kwath extract* showed maximum stimulation of TNFα levels in comparison to CHP *Kwath* extract and it was significant (p < 0.05). In all extracts, IFN-γ levels were comparable to Con A except marginally increase in *Kwath* of CRA and CHP HE extracts. The pretreatment of CRA and CHP extracts significantly inhibited IL-4 and IL-10 levels as compared to Con A (p < 0.05).

## 4 Discussion

In the last few decades, several plants are being validated as food and medicines. We have selected neglected species which are rarely documented, despite ethnic value as food/traditional medicines. Our institute has evaluated *O. sanctum (Tulsi), Ginger, Triticum aestivum (Wheat grass), etc*. for prebiotic*s* activity, antioxidant, anti-inflammatory and immunomodulatory roles [[22], [23], [24], [25], [26], [27]].

In the present study, attempts are made to validate the claims of *Patalagarudi* (*C. hirsutus*) and *Amarbel* (*C. reflexa*) having food-medicinal value, which is consumed by the local ethnic population. In *Materia Medica*, the description of CHP and CRA are documented as having immune rejuvenating properties [3]. Our effort was to assess antioxidant, anti-inflammatory, immune modulatory activities with mentioned plants in ethanolic, Hydroethanolic solvents apart from traditional (*Kwath* extract) preparation [[38], [39], [40]]. Recently, Singh et al., 2021 have assessed the Th1 and Th2 cytokines activity in *ex vivo* system with fresh juice, *Kwath* and alcoholic extracts of *Cynodon dactylon* and recorded the likely role of different preparation in various biological activities [24].

The CHP is a locally consumed green leafy vegetable, its compositional analysis was compared with *Amaranthus species, spinach leaves, cauliflower leaves*. Our institute periodically monitors the macro and micronutrient profiles of various food plants [32]. The presence of macronutrients (carbohydrates, proteins and fats), dietary fibers, Vitamins (B2, B3 and C) and minerals (Ca, K, P, Mg, Mn) of CHP was within ±10% variation in CHP. Gupta et al., 2005 report also endorses the presence of proteins, minerals, trace minerals and dietary fibers [41]. We are among the few to report compositional analysis of CRA and confirmed the presence of proximate principles and rich micronutrients like zinc, copper, iron, magnesium, selenium, manganese, etc. Hussain et al., 2009 [42] also confirmed such observations in CRA.

We have evaluated the biodynamic activities of unexplored plants to validate the folklore claims for health benefits. It was encouraging to note the presence of polyphenols in the extracts of CRA and CHP. Some previous studies also reported the presence of polyphenols viz., bergenin, chlorogenic, caffeic acid, and ferulic acid in CRA [14,43] and alkaloids such as Cohirsine, Cohirsinine, Jamtinine in CHP [[44], [45], [46]]. In the present study, it was also evident from UHPLC and UV spectra. The chromatogram peaks are present as “S” (strong), “M” (Medium), “L” (Low), and “T” (Trace) indicating the presence of polyphenols in different concentrations. As evident from UHPLC and UV spectra, the presence of caffeic acid, sinapic acid, ferulic acid, myricetin, quercetin, p-hydroxybenzoic acid, and chlorogenic acid was reported except protocatechuic acid in CHP. Protocatechuic acid was present as a strong peak but did not match to standard UV spectra. It is assumed, this could be due to any other substitution on the protocatechuic acid polyphenol. Further study is required to establish the fact.

The ellagic acid was present as a strong peak and matched to standard UV spectra absorbance in CRA. The chromatogram of daidzein (S) and bergenin (M) was evident on UHPLC without absorbance on UV spectra. The chromatograms of other polyphenols in the range of “M” to “T” viz., caffeic acid, Sinapic acid, ferulic acid, myricetin, and hesperetin in various extracts with the presence of absorbance on UV spectra were recorded.

As per the literature, many authors [47] have expressed the inability of assigning proper composition of polyphenols due to various reasons i. Concentration of polyphenols is very low since they are plant extracts, ii. Effect of different solvents and mediums used in extraction also make a great difference in elution pattern, iii. Polyphenols being conjugated systems, with different characteristics in different solvents generally show erratic UV absorption. Besides these, in the present study although an attempt was made to study the polyphenol patterns, it could not establish certain deviations in UHPLC and UV spectra, because of the fact that samples taken were crude extracts. Therefore, the scope of the present article remains open for further studies in determining the composition and structure of polyphenols using advanced studies in spectroscopy.

Chendouh et al., 2019 have reported a high concentration of phenols and polyphenols with different constituents along with antiradical activity (DPPH^·^ and ABTS^+·^) and inhibitory cell viability activity against neuroblastoma, hepatoblastoma, and colon carcinoma cells with Inula *viscosa* (unexplored plant) [31]. Our present investigation is a similar attempt, which confirms the significant concentration of TPC and diversity of polyphenols as evident by chromatograms and UV spectra of CRA and CHP extracts. The role of polyphenols like eugenol, curcumin, cinnamaldehyde present in clove, turmeric, cinnamon are established to have antioxidant, anti-inflammatory and immunomodulatory potential [27,48,49].

The observations from present study indicated dose-dependent free radical scavenging and inhibition activity with CRA and CHP extracts. The *Kwath* of CRA had maximum potential oxidative inhibition and free radical scavenging activity in comparison to other extracts. Raza et al., 2015 reported almost similar findings with the free radical scavenging potential of crude extract of CRA [50]. The use of the CRA juice in wound healing is documented which is attributed to the antioxidant potential. There are similar studies reporting the wound healing potential by virtue of their high content in antioxidant properties by various plants [51,52]. In the present study, the Eth and HE extract of CRA and CHP did not demonstrate potentially high dynamic activity in comparison to *Kwath* extracts.

Marvibaigi et al., 2016 demonstrated that phenolic compounds were the major contributors to antioxidant activity [53]. With respect to the presence of polyphenols we have demonstrated the presence of polyphenols in all extracts but could not establish the correlation to biodynamic activity. This is one of the limitation of this study.

The relationship between oxidative stress and immunomodulation is well known [54,55]. Many researchers have measured spleenocyte proliferation response through Th1and Th2 cytokines profiles [[22], [23], [24],^28^,^29^,^56^], thus validating immunomodulatory potential. Our observations are in line with these findings, demonstrating various extracts had immunomodulatory potential at various ranges.

One of the observations in this study is *Kwath* of CRA has immunomodulation in absence of Con A, indicative of mitogenic property. Similar observation was reported with *Aralia victoria, Boswellia carteri, Cyperus rotundus, Ginkgo biloba, Glycyrrhiza glabra, Olea europaea and Rosmarinus officinalis* [57]. Gautam et al., 2009 demonstrated the immunomodulatory activity of *A. racemosus* on systemic Th1/Th2 immunity and its implications for immuno-adjuvant potential [58]. CHP demonstrated Th1 immune stimulatory response, through up-regulation of TNFα and down-regulation of IL-4 and IL-10. Rastogi et al., 2008 also observed the immunostimulatory effect of CHP in cyclophosphamide-induced immunosuppressed rats [59]. Our observation on *Kwath* of CRA demonstrated up-regulation of TNFα and IFN-γ and down-regulation of IL-4 and IL-10 thus suggesting the ability to stimulate Th1 mediating immune response also. There are studies suggesting that CRA may be effective in host defense against tumor control, viral infection, and intracellular bacteria [14,15,60]. Our study findings are similar to the previous reports confirming the immunostimulating potential of *Dioscorea alata, Scrophularia variegate* [28,56]. In absence of relevant studies with CRA and CHP, our studies perhaps demonstrate immune response.

There are no relevant studies with CRA and CHP reported for its plausible role in Th1/Th2 immune response modulation. Gautam et al., 2009 have demonstrated that oral administration of *A. racemosus* (100 mg/kg) resulted in up-regulation of both Th1 (IL-2, IFN-g) and Th2 (IL-4) cytokines suggesting its mixed Th1/Th2 adjuvant activity [58]. The significance of the current study suggests the traditional formulation *Kwath* of CRA and CHP has potential immunomodulation and antioxidant activities.

The local population has the experience of prevention of various diseases by consuming the ethnic food grown in the area, which can be attributed due to enhanced immune functions. Similarly, in *Ayurvedic* text, the decoction of CRA has strong *Rasayana* properties, which we also confirmed in the present study. It is essential to note that the local information on medical and health treatment by traditional experience has significance and the validation further consolidates claims.

## 5 Conclusion

The current study validates immunomodulatory and antioxidant properties with the presence of phenolic content in the neglected plants CRA and CHP having ethnic food and medicinal value. This facilitates translating *Kwath* formulation of CRA and CHP as value-added nutraceuticals.

## Source of Funding

This work was supported by 10.13039/501100001411Indian Council of Medical Research-National Institute of Nutrition [15-FD05, 2015]. The fellowship support is given by University Grant Commission-JRF [1391/(NET-DEC.2012)].

## Author contributions

**Anita Singh:** Conceptualization, Methodology, Data curation, Writing - Original draft preparation. **Vandana Singh**: Conceptualization, resources, review and editing. **Dinesh Kumar**: Supervision, Conceptualization, Project administration. **Ananthan**: Methodology.

## Conflict of interest

None.
