# Supplementary material for: Evaluation of immunomodulatory and antioxidants properties of Kwath, conventional extracts in plants Cocculus hirsutus and Cuscuta reflexa – in vitro & ex vivo studies
Source: J Ayurveda Integr Med. 2022 Jan 10;13(1):100537. doi: 10.1016/j.jaim.2021.100537 (PMC8760447; doi:10.1016/j.jaim.2021.100537)
Supplement: Multimedia component 1 [file mmc1.docx]

Supplementary data


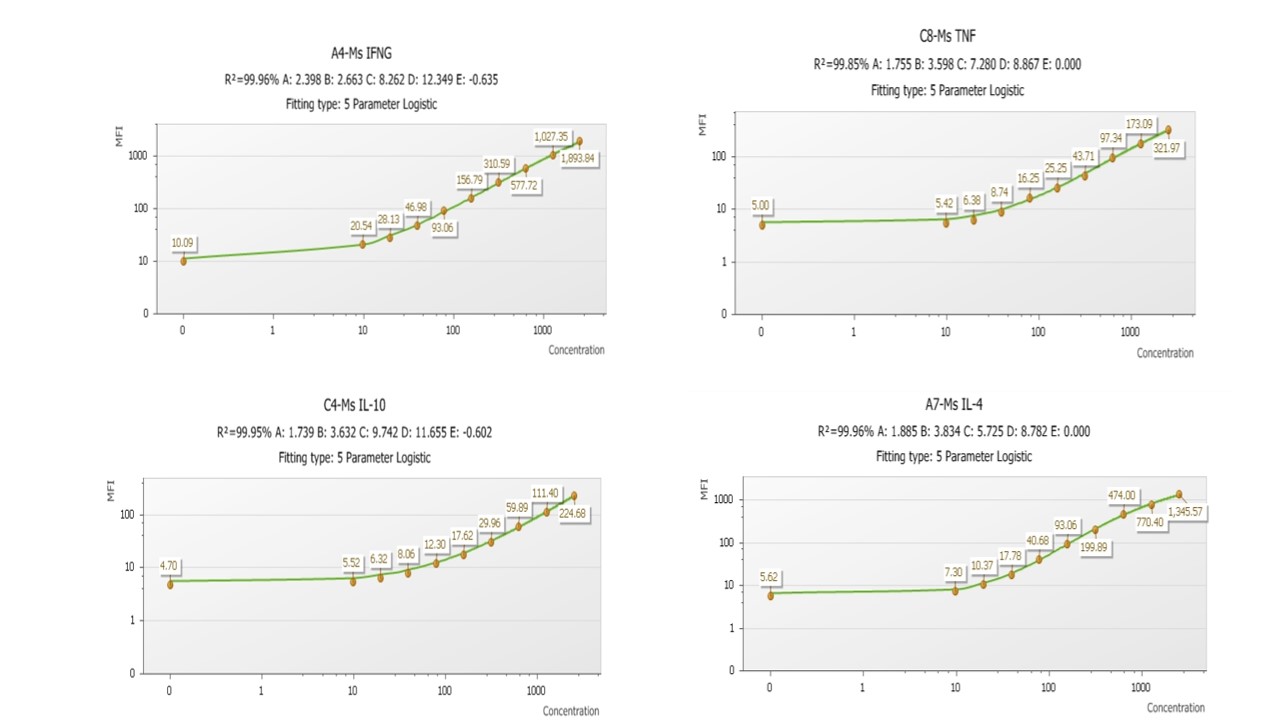


Fig S1: Th1 and Th2 cytokines standard curves generated by BD CBA Software (FCAP) after acquiring data from flow cytometer .The standard curves (0 pg/ml to 2500 pg/ml) were plotted for all four cytokines; IFN-gamma, TNF, IL-10 and IL-4 (concentration vs. mean fluorescence intensity; MFI) using a five-parameter logistic curve fitting model


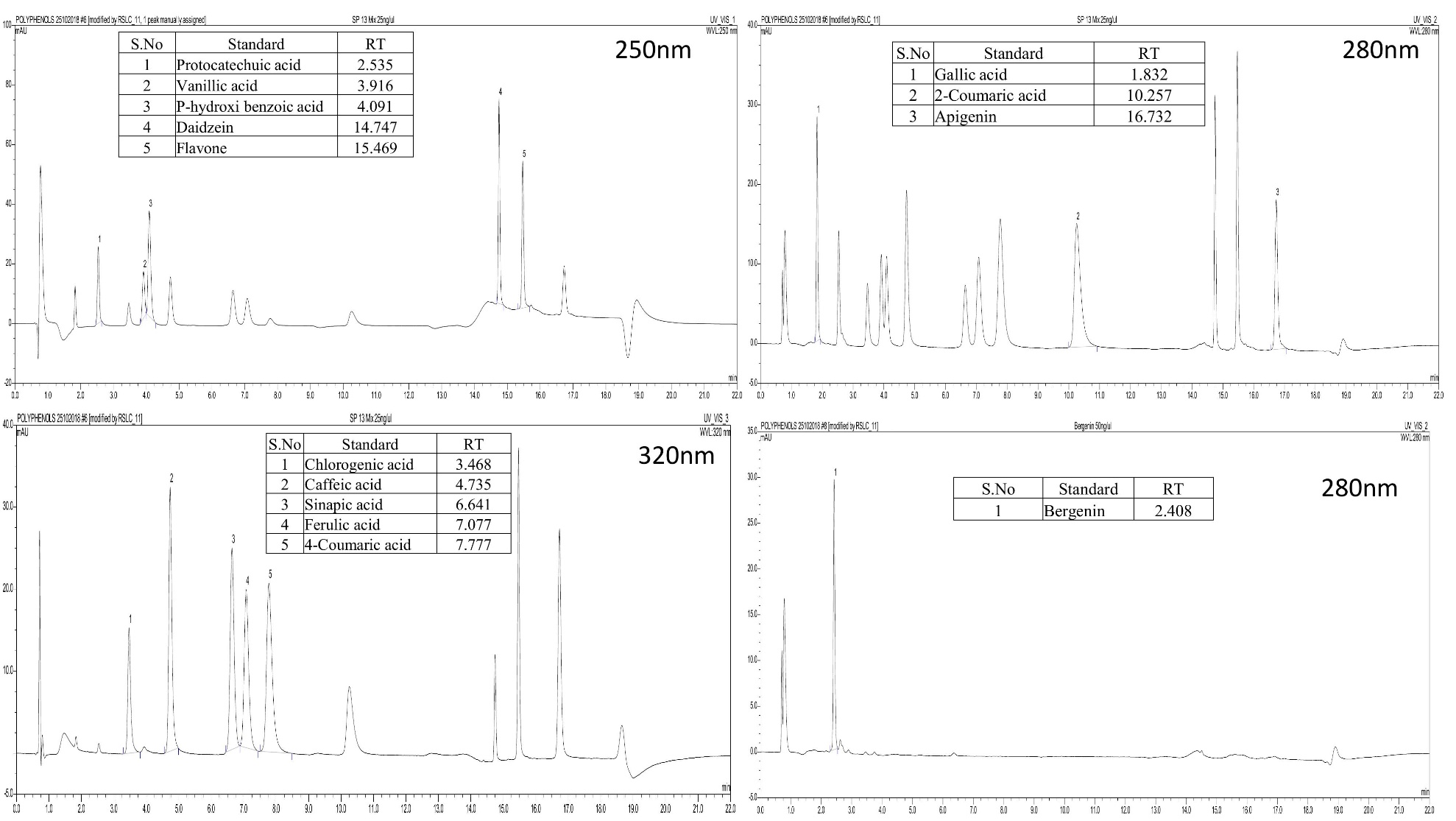


Fig S2: U High-Performance Liquid Chromatography (UHPLC) of standard mixture of polyphenols (Gallic acid, Protocatechuic acid, Chlorogenic acid, Vanillic acid, p-hydroxy benzoic acid, Caffeic acid, Sinapic acid, Ferulic acid, 4-Coumaric acid, 2-Coumaric acid, Apigenin, Daidzein and Bergenin) with retention time (RT).


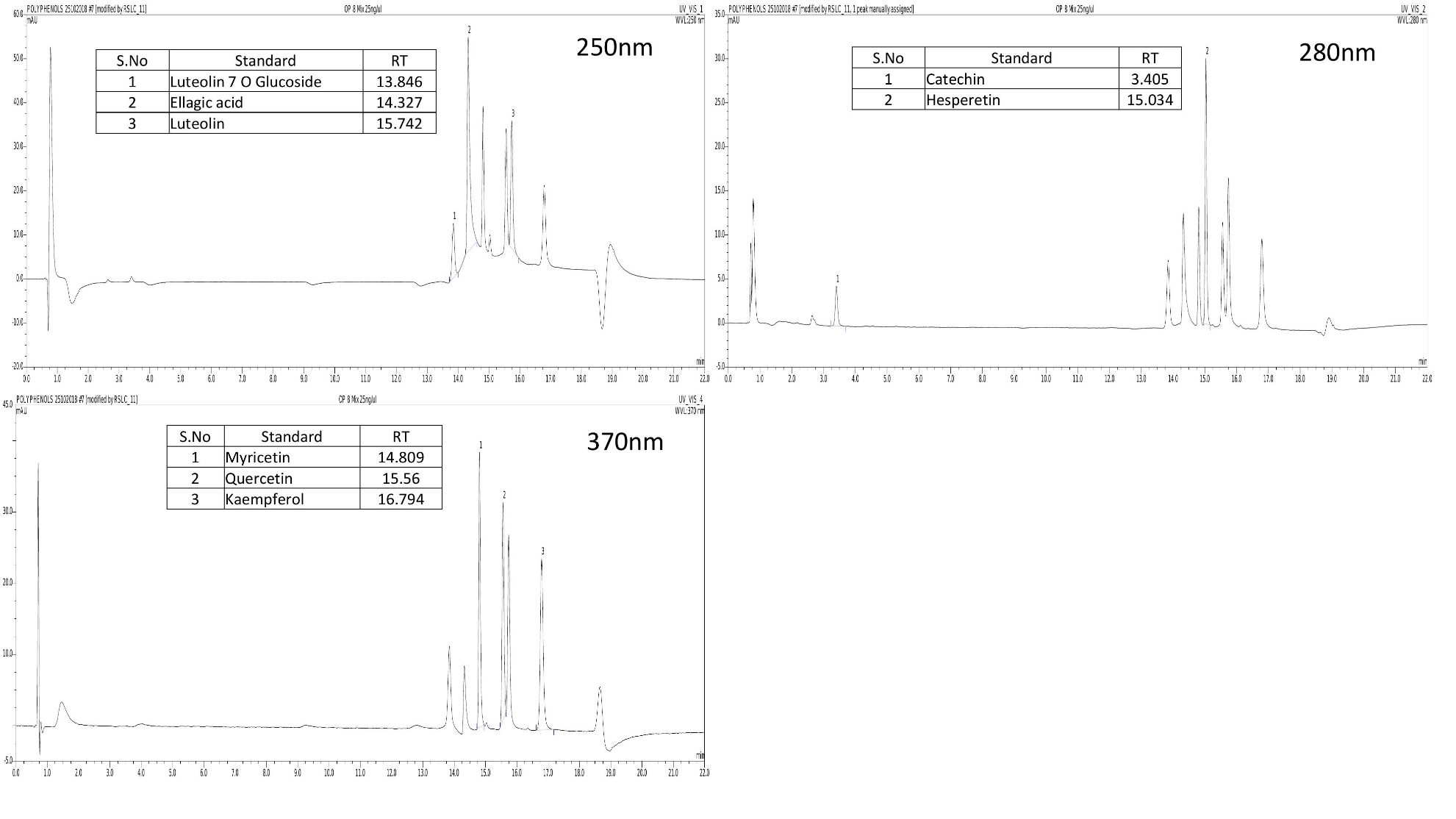


Fig S3: U High-Performance Liquid Chromatography (UHPLC) of standard mixture of polyphenols (Catechin, Luteolin7 O Glucoside, Ellagic acid, Myricetin, Hesperetin, Quercetin, Luteolin and Kaempferol with retention time (RT).


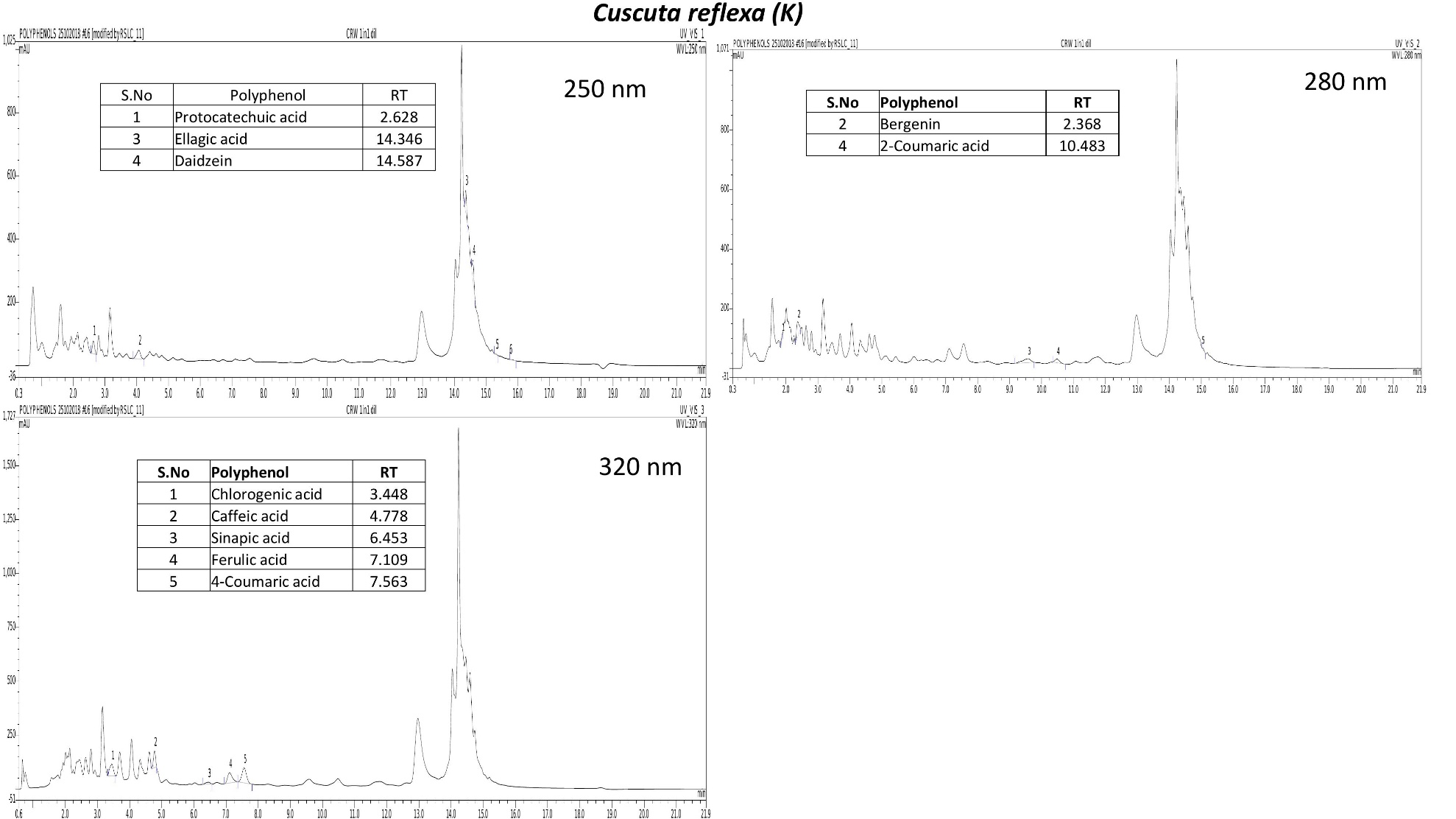


Fig S4: U High-Performance Liquid Chromatography (UHPLC) of *Cuscuta reflexa* (Kwath).


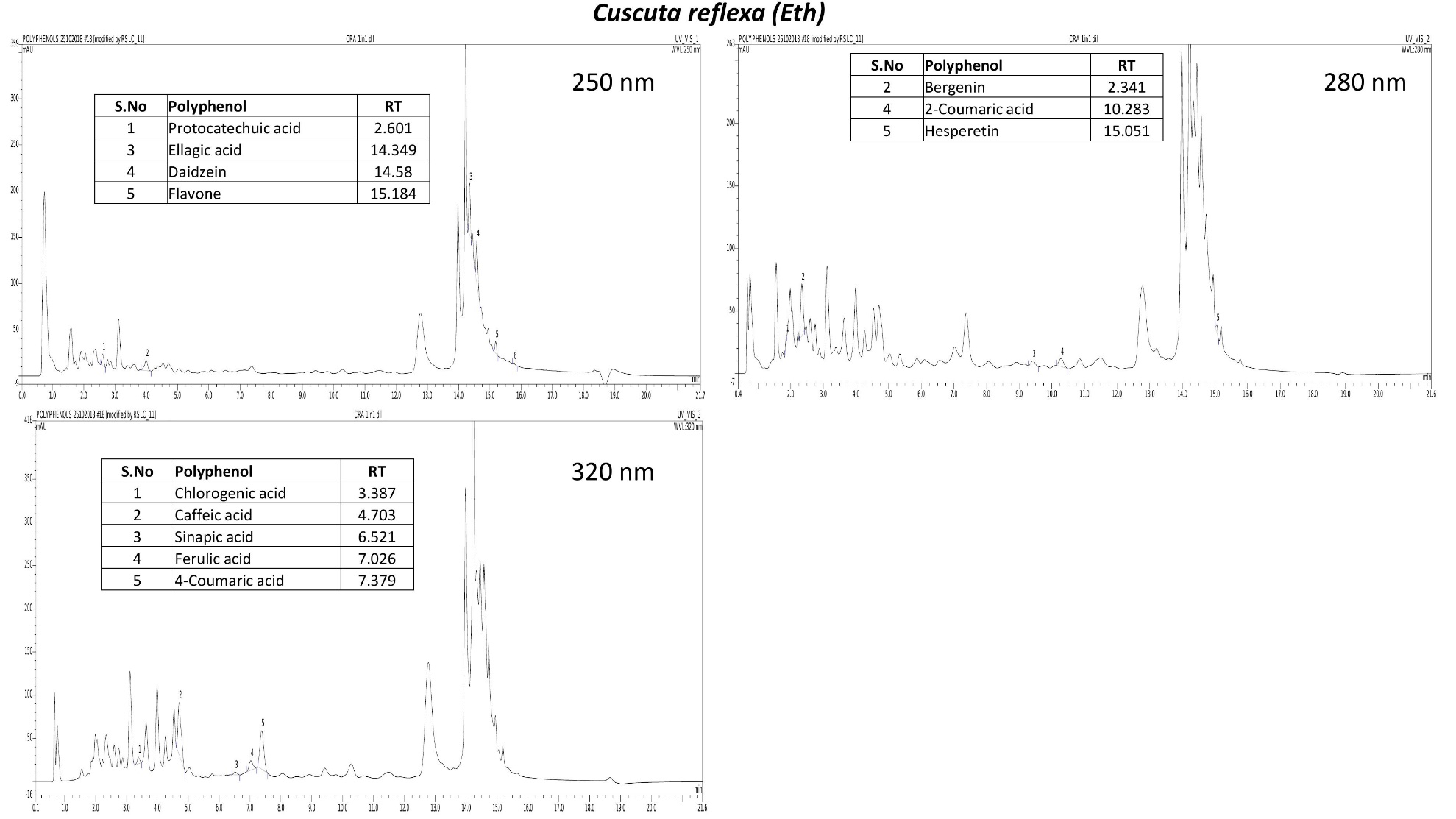


Fig S5: U High-Performance Liquid Chromatography ( UHPLC) of *Cuscuta reflexa* (Ethanolic).


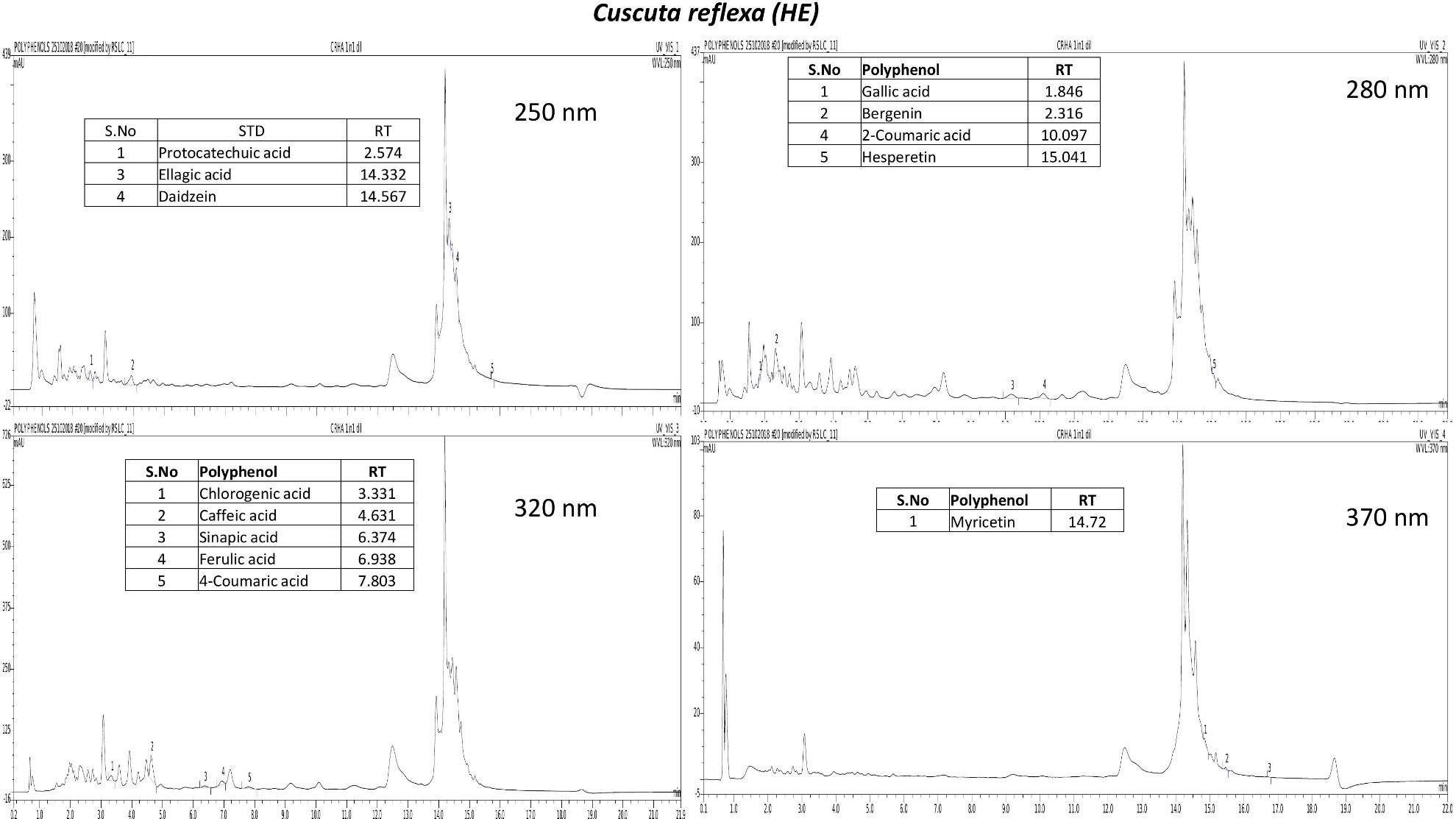
Fig S6: U High-Performance Liquid Chromatography (UHPLC) of *Cuscuta reflexa* (Hydro-ethanolic).


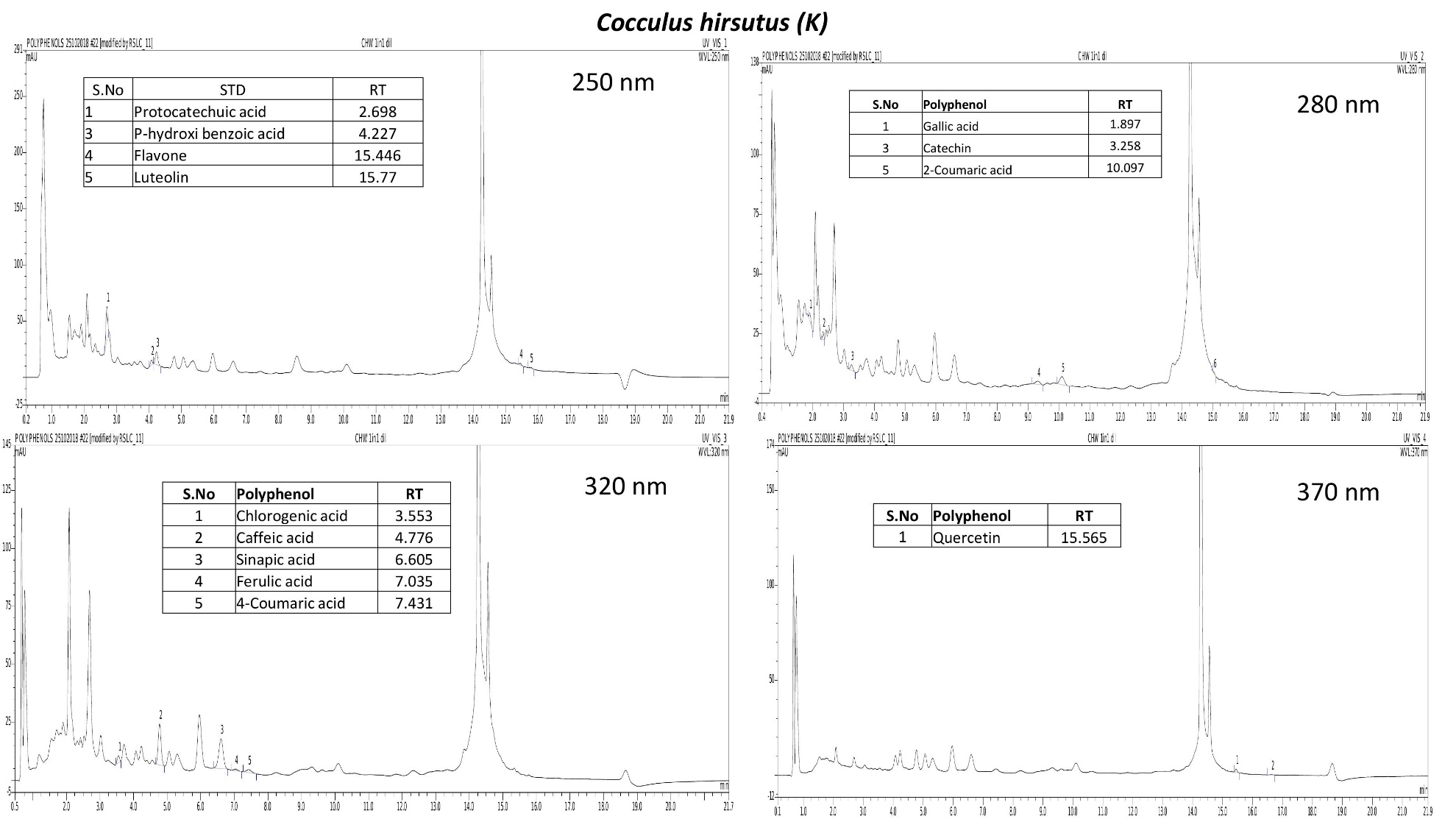


Fig S7: U High-Performance Liquid Chromatography (UHPLC) of *Cocculus hirsutus* (Kwath).


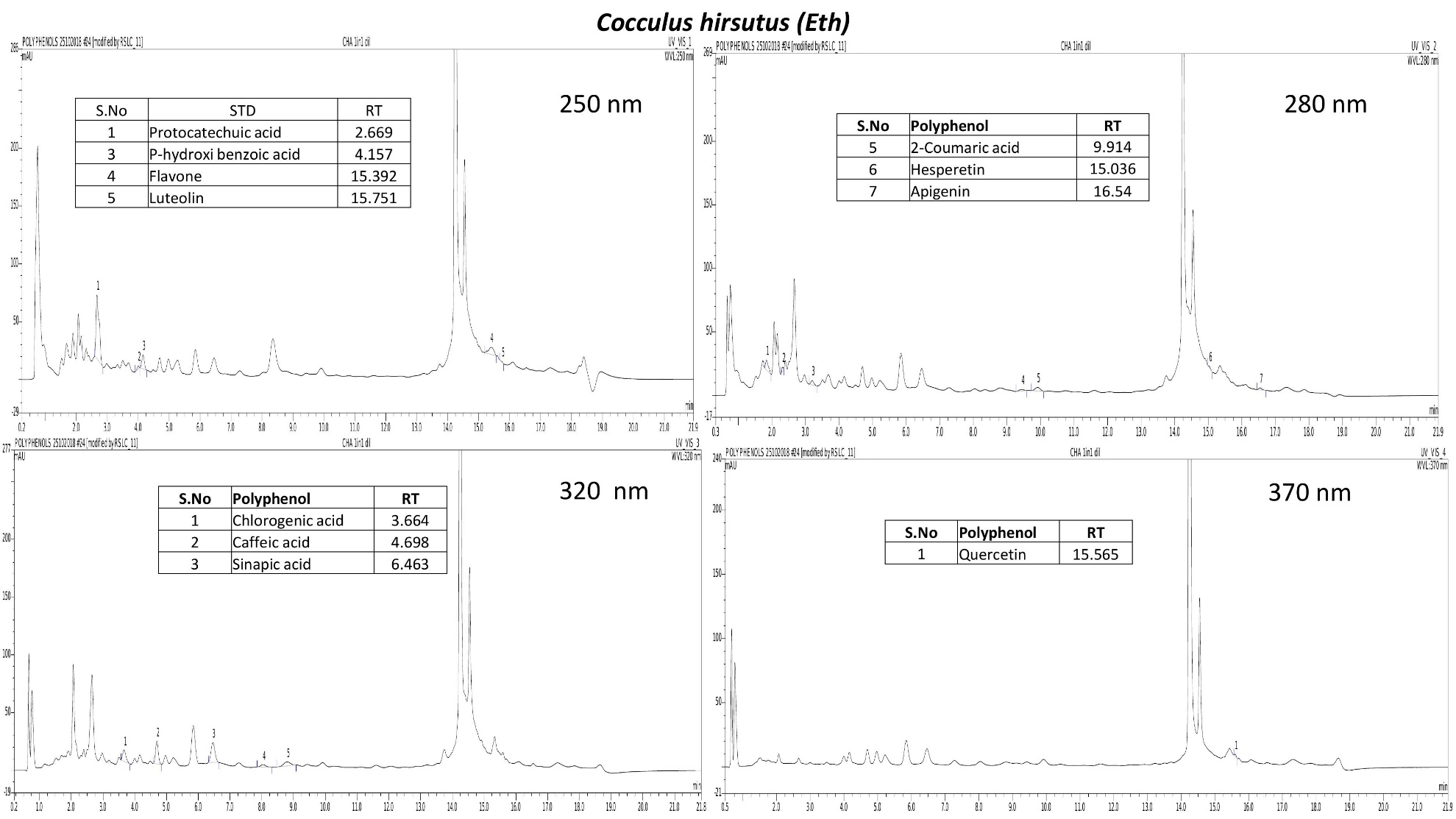


Fig S8: U High-Performance Liquid Chromatography (UHPLC) of *Cocculus hirsutus* (Ethanolic).


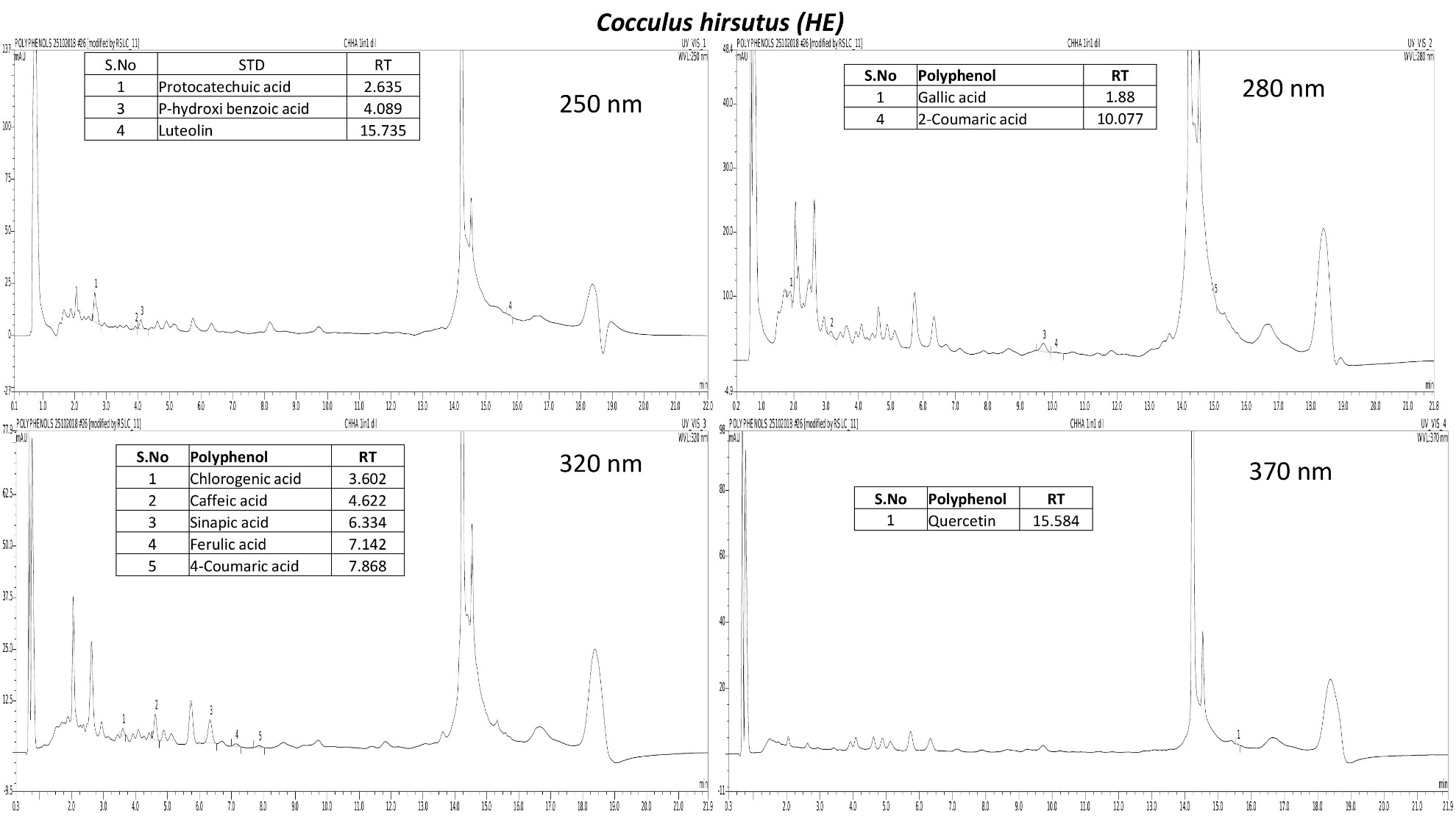


Fig S9: U High-Performance Liquid Chromatography (UHPLC) of *Cocculus hirsutus* (Hydro-ethanolic).

Fig S10a: Extracted spectrum with extinction coefficient of different polyphenols components in the standard (black line spectra) as well as samples (red color spectra).

***a)Cuscuta reflexa* (Kwath)**

Fig S10b: Extracted spectrum with extinction coefficient of different polyphenols components in the standard (black line spectra) as well as samples (red color spectra).

***b) Cuscuta reflexa* (Ethanolic)**

Fig S10c Extracted spectrum with extinction coefficient of different polyphenols components in the standard (black line spectra) as well as samples (red color spectra).

***c) Cuscuta reflexa* (Hydro-ethanolic)**

Fig S10d: Extracted spectrum with extinction coefficient of different polyphenols components in the standard (black line spectra) as well as samples (red color spectra).

**d)** ***Cocculus hirsutus* (Kwath)**

Fig S10e: Extracted spectrum with extinction coefficient of different polyphenols components in the standard (black line spectra) as well as samples (red color spectra).

 e) ***Cocculus hirsutus* (Ethanolic)**

Fig S10f: Extracted spectrum with extinction coefficient of different polyphenols components in the standard (black line spectra) as well as samples (red color spectra).

**f) *Cocculus hirsutus* (Hydro-ethanolic)**
